# Measuring what matters in healthcare: a practical guide to psychometric principles and instrument development

**DOI:** 10.3389/fpsyg.2023.1225850

**Published:** 2023-09-18

**Authors:** Katina Swan, Renee Speyer, Martina Scharitzer, Daniele Farneti, Ted Brown, Virginie Woisard, Reinie Cordier

**Affiliations:** ^1^Curtin School of Allied Health, Curtin University, Perth, WA, Australia; ^2^St. John of God Midland Public and Private Hospitals, St John of God Health Care, Perth, WA, Australia; ^3^Department of Allied Health, The School of Medical and Health Sciences, Edith Cowan University, Perth, WA, Australia; ^4^Department Special Needs Education, University of Oslo, Oslo, Norway; ^5^Department of Otorhinolaryngology and Head and Neck Surgery, Leiden University Medical Centre, Leiden, Netherlands; ^6^Department of Biomedical Imaging and Image-guided Therapy, Medical University of Vienna, Vienna, Austria; ^7^Audiologic Phoniatric Service, Otorhinolaryngology Department, Infermi Hospital, AUSL Romagna, Rimini, Italy; ^8^Department of Occupational Therapy, Faculty of Medicine, Nursing and Health Sciences, Monash University – Peninsula Campus, Frankston, VIC, Australia; ^9^Centre Hospitalier Universitaire de Toulouse, Touloluse, France; ^10^Department of Social Work, Education and Community Wellbeing, Northumbria University, Newcastle upon Tyne, United Kingdom; ^11^Department of Health and Rehabilitation Sciences, Faculty of Health Sciences, University of Cape Town, Cape Town, South Africa

**Keywords:** psychometrics, measurement, instruments, COSMIN, validation, instrument development

## Abstract

The provision of quality healthcare relies on scales and measures with robust evidence of their psychometric properties. Using measurement instruments with poor reliability, validity, or feasibility, or those that are not appropriate for the target diagnostic group or construct/dimension under consideration, may be unfavorable for patients, unproductive, and hinder empirical advancement. Resources from the COnsensus-based Standards for the selection of health status Measurement INstruments (COSMIN) group can assist in identifying and developing psychometrically sound measures. The COSMIN initiative is the only international, research-based practice taxonomy and methodological guidelines for measurement in healthcare. This manuscript aims to provide an accessible introduction to theories, principles and practices of psychometrics, instrument properties, and scale development, with applied examples from the COSMIN recommendations. It describes why measurement in healthcare is critical to good practice, explains the concepts of the latent variable and hypothetical construct and their importance in healthcare assessments, explores issues of flawed measurement and briefly explains key theories relevant to psychometrics. The paper also outlines a ten-step process to develop and validate a new measurement instrument, with examples drawn from a recently developed visuoperceptual measure for analysis of disordered swallowing to demonstrate key concepts and provides a guide for understanding properties of and terminology related to measurement instruments. This manuscript serves as a resource for healthcare clinicians, educators, and researchers who seek to develop and validate new measurement instruments or improve the properties of existing ones. It highlights the importance of using psychometrically sound measurement instruments to ensure high-quality healthcare assessments.

## 1. Introduction

Measurement is integral to clinical practice and evidence-based healthcare. Measurement may be defined as the systematic and orderly categorization of the attributes of an object, phenomenon or concept that creates a shared standard (Bond et al., 2020). As a fundamental activity of science, it serves as a foundation for evidence-based practice (McClimans, 2017; Bond et al., 2020). The journey toward evidence-based medicine in healthcare runs in parallel with progress toward effective measurement, as quantifiable evidence underpins evidence-based care. Accurately quantifying and categorizing observations is vital for attributing clinical meaning to data (Streiner et al., 2015). Strong measurement relies on sound practices in instrument development and validation (Streiner et al., 2015). However, many clinicians, particularly in healthcare disciplines without strong psychology components, may lack training in psychometrics to engage with instrument validation science. Our manuscript seeks to address this gap by presenting concepts, pathways, and applied knowledge in a manner that is easily understandable and applicable for clinicians, and for educators and researchers working in healthcare who have an interest in psychometrics.

### 1.1. Manuscript structure and aim

**Figure 1:**
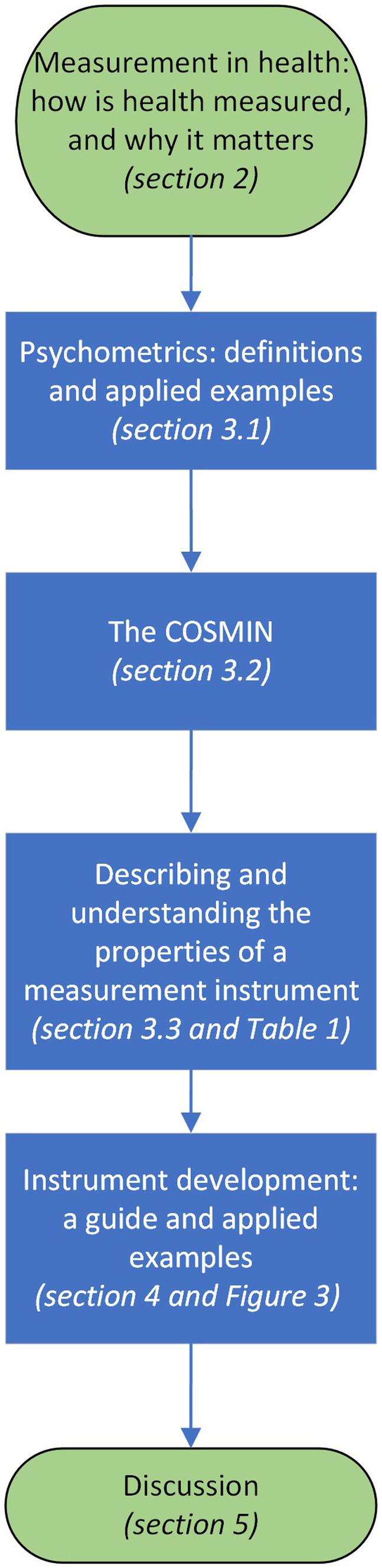
Manuscript Structure.

This manuscript aims to introduce key principles of psychometrics for healthcare clinicians from medical, nursing and allied health backgrounds and healthcare educators and researchers who are interested in understanding the overall topic measurement and scales, tests and instruments used to measure constructs, factors, attributes, or dimensions. It aims to empower clinicians to navigate psychometrics research, understand relevant criteria and support application of good psychometrics practices to clinical instrument development and selection. Figure 1 outlines the structure of the manuscript, which will describe psychometric terms, introduce the Consensus-based Standards for the selection of health status Measurement Instruments (COSMIN) (Mokkink et al., 2018), present core concepts in measurement, define and provide introductory guidance on understanding the properties and quality of a measurement instrument. The manuscript will also deliver a guide for key steps in measure development through the applied example of a recently developed instrument for difficulty swallowing, also known as dysphagia.

## 2. Measurement in health

In healthcare, concepts of measurable interest may be considered to be a pin-point or *ballung* (McClimans, 2017). A pin-point measurement is a discreet category against which a patient may be compared over time, such as weight, age, and mortality. *Ballung* concepts, which owe their name to the German word “Ballungsgebiet” (English: agglomeration), which refers to a congested urban area with unclear borders, are more challenging to define and articulate (McClimans, 2017). Considering real clinical measurement challenges can lead to the conclusion that most health concepts are *ballung.* Box 1 outlines an applied example.

**Table 1:** COSMIN preferred statistical analysis with conceptual examples.

	Preferred statistical analysis	Non-statistical preferred analysis method	Purpose/implications
Content Validity	N/A	It comprises three aspects: Relevance (the extent to which the items are appropriate and relevant to the target construct), comprehensiveness (whether the instrument covers all aspects of the construct), and comprehensibility (whether the items are easy to understand and interpret by the users). Quality criteria should be applied to judge the quality of the study methods to verify a scale’s content validity (Mokkink et al., 2019).	Demonstrates relevance and comprehensiveness of the instrument to the target construct that is ‘in-built’ from instrument development methodology (Vetter and Cubbin, 2019). Content validity is considered the most important psychometric property (Mokkink et al., 2019). It should have been established in the early stages of creating the scale with sound methodology (e.g., Delphi study or literature review for deductive method, observation, or patient focus groups for inductive approach). Poor content validity means the entire instrument may be of questionable use. Content validity ensures that the items of a scale or instrument represent or reflect the latent target variable being measured.
Face validity	N/A	Examination by professionals and patients (Mokkink et al., 2019)	Face validity demonstrates the extent to which informed stakeholders judge that the items that make up an instrument appear to be representative of the target construct of interest at a surface level (e.g., experts with clinical knowledge of the target disease, patients with lived experience of the condition of interest) (Vetter and Cubbin, 2019). In other words, the items that comprise an instrument appear at ‘face value’ to reflect the latent target variable. An advantage of face validity testing is the relative ease and speed of assessment compared to other psychometric aspects; however, as it is assessed subjectively, it may be biased.
Construct Validity			Construct validity describes the relationship between the instrument and other instruments or outcomes of interest. Poor construct validity may imply that the instrument is accessing a different construct compared to similar instruments.
Structural validity^*^	CTT**: Exploratory Factor Analysis and Confirmatory Factor Analysis IRT: Model fits the research question (Mokkink et al., 2019).		Demonstrates that all items in the instrument ‘belong’ in the instrument; that is, they all access the same underlying construct. The items should demonstrate some relationship to each other (correlation) but may be able to be grouped to demonstrate that they assess discrete dimensions of the same construct. In CTT, exploratory factor analysis (EFA) identifies the different factors (or dimensions) that the items may be able to be grouped into. In the instrument development phase, EFA will often result in some items being removed, as they do not fit into any identified factors. It is also possible that the items of a scale that load onto a factor may not make conceptual sense and, therefore, may be discarded. Just because the items in an EFA load on a factor does not infer that they will be automatically kept. The retained items may form subscales in the subsequent version of the instrument. Confirmatory factor analysis (CFA) tests the hypothesized factors’ presence and structure. CFA involves specifying a model of the factor structure, including the number of factors, the relationships between factors, and the relationships between the factors and items. This information is based on the existing literature and theories about the construct being measured. Both EFA and CFA should be performed in the instrument development and validation phase, with EFA occurring before CFA. EFA is a preliminary exam that identifies factor structures and relationships, allowing the developer to explore the data without making any assumptions or hypotheses about the number of factors or the relationships between variables. Based on the results of the EFA, the developer can then make informed decisions about the factor structure to be tested in the CFA. By conducting an EFA first, the developer can ensure that the model specified in the CFA is a good representation of the target latent variable/factor and is not limited by any prior assumptions or hypotheses about the factor structure (Yong and Pearce, 2013; Brown, 2015). EFA and CFA will also report on the amount of variance explained by these factors (expressed as a percentage). Factor analysis examines the interconnections between items. Variance describes how much the individual scores in a population vary from the mean group score (i.e., dispersion of values from the average score). Factor analysis that explains > 70% of the variance is preferred (Hinkin, 1998; Mokkink et al., 2018), as it demonstrates that the model accounts for most of the spread from average (i.e., that most items are inter-correlated).
Hypothesis testing	Statistical methods appropriate to the hypothesis (Mokkink et al., 2019)		Hypothesis testing is an expression of whether the instrument behaves in the way developers expect. These hypotheses may include: convergence, which refers to the extent to which multiple instruments of the same construct produce similar results that are positively correlated; divergence, which refers to the situation where multiple instruments of the same construct produce differing results that are not correlated or are negatively correlated with each other; and diagnostic accuracy, which assesses the ability of the instrument to identify individuals with the target disorder accurately. For example, they may hypothesize that instrument A will result in a very similar score to instrument B (evidenced by positive correlation) and that there will be no significant difference in scores between population C and D. Developers must choose the appropriate statistical method to test their hypothesis – for example, Pearson’s R correlation coefficient to demonstrate the correlation of instrument A and B, or a Mann–Whitney U to explore the difference in two populations.
Cross cultural validity	CTT: Regression analyses or multi-group confirmatory factor analysis (MGCFA) IRT: IRT/Rasch-based analyses: ≥200 patients per group, differential item functioning analysis (Mokkink et al., 2019)		Cross-cultural validity demonstrates whether an instrument from one population is valid in a new population. Validity can be investigated using several methods. Regression analyses can examine the relationship between the instrument scores and potential population variables, such as language, socioeconomic status, quality of life, or sociocultural values. This helps to determine if the instrument measures the same construct across both participant groups. MGCFA tests if the instrument produces a similar pattern of results across both target populations (Brown et al., 2017). The factor loadings can be constrained to fit the same loadings the original population produced. The equivalence (aka measurement invariance) and change in the fit of these loading between the populations assessed provide evidence of comparable structural validity across groups. MGCFA tests if the instrument is biased in one group (Pendergast et al., 2017). Differential item functioning fullfils a similar purpose, with analysis testing if the items work the same way in different populations (can also be considered the ‘fairness’ of the items); lack of invariance is considered evidence of equivalent performance (Hagquist and Andrich, 2017). Reliability tests (e.g., internal consistency, test–retest) and hypothesis testing for convergent validity may also be undertaken.
Criterion Validity	Continuous scores: correlations, or the area under the receiver operating curve (AUC) Dichotomous scores: sensitivity and specificity (Mokkink et al., 2019)		Criterion validity demonstrates the relationship between the instrument under analysis and the ‘gold-standard’ (best available measure under reasonable conditions, that is, context or environment optimal for good measurement). The gold-standard is the benchmark against which other instruments should be compared. Correlations demonstrate the strength of associations between two scores, with-1 indicating a negative association, 0 indicating no association, and + 1 indicating a positive association. Generally, 0.90–1.00 is considered a very high correlation, and 0.70–0.89 is high. However, for a large sample, a lower correlation score is typically considered statistically acceptable (Asuero et al., 2006). Sensitivity is the instrument’s ability to correctly identify the target construct (e.g., the presence of disease). Specificity is the instrument of accuracy in identifying the absence of the construct (e.g., free of disease). They are calculated in a 2×2 table, where: sensitivity = How well does the screen/instrument test for the presence of disease? Calculated with (true positive)/(true positive + false negative); produces a probability of being tested positive when the disease is present. specificity = How well does the screen/instrument test for the absence of disease? Calculated by (true negative)/(true negative + false positive); produces the probability of being test negative when the disease is absent. They are inversely proportional; as one increases, the other will fall. (Parikh et al., 2008). The acceptable percentage will vary depending on the intended purpose of the instrument; for a high-stakes disease (i.e., high mortality) with a low-stakes outcome, higher sensitivity may be preferable (e.g., cancer, where the next step in the exam would be a simple blood test to confirm). However, higher specificity would be preferred in a lower stake disease or a higher stakes outcome (e.g., esophageal dysmotility, where the next step in the investigation could be endoscopy requiring a general anesthetic). This is because it is preferable to ‘net’ more patients if the disease is high stakes, even if it means increasing false positives. If the disease is low stakes, but the treatment or investigation is highly invasive, false positives may be undesirable.
Internal Consistency	Continuous scores: Cronbach’s alpha or Omega for each unidimensional scale or subscale Dichotomous scores: Cronbach’s alpha or KR-20 for each uni-dimensional scale or subscale IRT-based scores: standard error of theta (SE (θ)) or reliability coefficient of estimated latent trait value (index of [subject or item] separation) for each unidimensional scale or subscale (Mokkink et al., 2019)		Internal consistency demonstrates the strength of relationships among items. Cronbach’s alpha or omega describes inter-item covariance, or how item A changes when item B changes. Scores usually range from 0–1.0, but a negative α value can occur when the items are not positively correlated. An alpha value of 0.70–0.80 is often considered “adequate” for human sciences, but the value can be artificially inflated in instruments containing many items (49). An adequate or higher range of Cronbach’s alpha or omega indicates that the items are all related to each other (measuring a similar construct). Too high, and there may be redundancy (two or more items assessing the same aspect of a construct); too low indicate item(s) are irrelevant or ill-fitting. The standard error of theta fullfils a similar function to Cronbach’s alpha or omega in identifying ill-fitting items but uses a different statistical process. It instruments the relationship between an item and the population mean (i.e., the average scores achieved on the instrument vs item scores). Items that demonstrate a weak relationship may be ill-fitting.
Reliability	Continuous scores: Intraclass Correlation Coefficient (ICC); Ordinal scores: weighted Kappa; Dichotomous/nominal scores: Kappa calculated for each category against the other categories combined (Mokkink et al., 2020)		Reliability indicates the stability across time or between raters/within patients. ICC and weighted Kappa indicates the similarity between two groups of scores (e.g., one taken by rater A and one by rater B). 0 indicates nil or low similarity, while 1.0 indicates perfect agreement. Generally, values of > 0.75 are considered strong (Portney and Watkins, 2009). COSMIN quality criteria consider an ICC or weighted Kappa of 0.70 sufficient (Prinsen et al., 2018). It should be noted that reliability statistics are not evidence of validity; reliability shows that the instrument achieves the same score if used by different people or at different times. It does not indicate the relationship of the instrument to the latent construct.
Measurement Error	Continuous scores: Standard Error of Measurement (SEM), Smallest Detectable Change (SDC), Limits of Agreement (LoA) or Coefficient of Variation (CV) (Mokkink et al., 2020)	Dichotomous/nominal/ordinal scores: the percentage specific (e.g., positive and negative) agreement	Analysis of measurement error assesses agreement. Measurement error refers to the difference between a variable’s true value and estimated value resulting from inaccuracies in the measurement process, such as instrumentation error, observer bias, or subject variation (Hernaez, 2015). SEM is the standard deviation of measurement errors associated with scores from a specific population. This can then create ‘confidence bands’ around scores; that is, the likelihood that an accurate score falls within a specific range. SDC is the minimum changes in scores for the developer to the confident the change is real (not introduced error or random error). SDC can be calculated for individual patients and compares group means. Taken together, SEM and SDC provide information about the reliability (stability) of the instrument by indicating the boundaries wherein the true score lies and describing the scale of change in scores required before the developer can be certain change is real (Geerinck et al., 2019). Developers may also examine Minimal Important Change (MIC) – the smallest change in a test score representing a meaningful and clinically significant difference for an individual. SDC should be smaller than MIC so that changes in scores that are meaningful and important to patients and clinicians can be reliably detected (de Vet and Terwee, 2010). LoA likewise describes the boundaries for the scope of range wherein the true score may lie and is calculated using the overall mean difference in the test and retest scores (Polit, 2015). The CV also measures the spread of scores around the mean by calculating the ratio between the standard deviation and the mean, expressed as a percentage. Generally, a lower score is better, as it means less error in the instrument and a smaller range wherein the true score may lie (Riemann and Lininger, 2018).
Responsive-ness	Idem criterion validity; For dichotomous scores: sensitivity and specificity (changed versus not changed)		Correlations describe the degree of association between the instrument and the gold standard, but with the addition of repeated use of the instrument, track change over time (i.e., the correlation between change over time in the instrument and change over time in the gold standard). A score of 1.0 would imply a perfect correlation with the gold standard. The COSMIN considers results in accordance with the hypothesis, correlation of 0.70 or (for sensitivity/specificity) AUC of > 0.70 sufficient (Prinsen et al., 2018).

^*^Only relevant for measures based on a reflective model. ^**^CTT, Classical Test Theory.

BOX 1 **The multidimensional nature of health phenomena: coronary artery disease.**

The physician interested in tracking a patient with coronary artery disease may choose from a number of aspects to measure; biometrics, such as cardio-respiratory function, haematological markers, electrocardiogram signals or images from coronary angiography (Gillen and Goyal, 2022). Coronary artery disease has a negative effect on health-related quality of life and limits exercise tolerance, while angor amini, a sense of impending doom, is a well-known symptom of acute coronary syndrome and may also be worthy of checking (Goldbeck-Wood, 2019; Huzmeli et al., 2020). Therefore, to complete the clinical picture, the physician may need to gather data from a range of facets. This example shows how a single diagnosis may have multiple clinically relevant variables of interest to the physician.

Dimensions are aspects of a construct; for example, health-related quality of life in diabetes may be impacted by fatigue, reduced participation or changed family roles (De Vet et al., 2011). In healthcare measurement science, the concept of ‘multidimensionality’ acknowledges that many health constructs cannot be comprehensively captured by a unidimensional measure alone. The challenge of accurately capturing the full range of function and dysfunction of *ballung,* or multidimensional, concepts becomes even more complex in disorders that are not yet fully understood or have a range of clinical presentations (e.g., rare neurological diseases or multifactorial genetic conditions). This can lead to bias or incomplete representation. Despite this, measurement instruments are essential in healthcare as they establish intervention goals, provide comparison baselines, aid diagnosis, monitor changes and intervention impact, and track efficacy and cost–benefit. Indeed, it has been asserted that ‘you cannot manage what you cannot measure’(McClimans, 2017). Therefore, faced with complex constructs and the impossibility of continuous measurement in the context of healthcare’s finite time and resources, clinicians and healthcare professionals must carefully consider what, when, where, and how to measure. Appropriately designed and statistically robust measurement instruments are therefore essential. Understanding key terminology and concepts in instrument design and function improves measurement selection, supporting better clinical practice and research.

### 2.1. The structure and function of a measurement instrument

In healthcare, various types of measures are used such as surveys, questionnaires, assessment booklets with matching stimuli, specialty devices, and analysis checklists. All of these fall under the category of measurement instruments, measures, or scales. Throughout this manuscript, the term ‘measurement instrument’ or ‘instrument’ will be used to refer to all these concepts for the sake of clarity. Measurement instruments in healthcare can be classified into two broad categories: clinician-reported measures and patient-reported outcome measures (PROMs). Clinician-reported measures are based on the observations, ratings, and assessments made by the clinician. In contrast, PROMs are measurement instruments based on the perspectives and experiences of patients, family members/caregivers, or others affected by the target issues. PROMs are designed to capture the patient’s subjective view of their health status, function, symptoms, and quality of life (De Vet et al., 2011).

Measurement instruments can be further divided into three components: domains, items and response scales (Supplementary Figure S1 describes an applied example). The *domain* is the content area under investigation, the *items* are the aspects of the domain that are of interest, and the *response scales* are the organization of the items into a measurable form (i.e., orderly and unambiguous description or marker of the degree to which the domain is present) (Streiner et al., 2015; Lambert and Newman, 2022). Response scales come in various forms – for example, they may be continuous, dichotomous, ordinal, nominal or interval (Streiner et al., 2015). One of these may be superior, depending on the question the clinician seeks to answer and the construct or dimension under examination. The construct may be considered the core of the instrument. It is the well-defined hypothetical phenomenon, dimension, attribute, factor or concept that the clinician or researcher is interested in measuring (De Vet et al., 2011). It may or may not be possible to observe the construct or dimension directly.

### 2.2. The hypothetical construct

Most of what is assessed in healthcare is a latent variable reliant on a proxy which is then translated into numerical values. In strict terms, the numbers on a thermometer are not a measure of body temperature. They measure the amount of electrical voltage passed through metal electrodes, which changes depending on how warm or cool the metals are. The numbers of a scale, Celsius or Fahrenheit, are then applied to the resulting resistance, which correlates to the latent variable – human body temperature.

Since it is not feasible or possible to directly observe the latent variable, the challenge in measurement is determining how well the observable maps onto the latent variable (Sechrest, 2005). The issues that these present are outlined in the example in Box 2.

BOX 2 **The latent variable: blood pressure and the sphygmomanometer.**

Blood pressure can be measured in various ways/positions (standing, sitting, supine, upper arm, ankle) and using different methods (gradient, diastolic/systolic pressure difference) with the application of a pressure cuff (sphygmomanometer). However, the relationship between the instrument's technical properties and the construct (blood pressure) is not as straight-forward as may be assumed. The sphygmomanometer does not directly measure blood pressure but instead uses a strain gauge to convert mechanical pressure into a rise or fall of liquid in a tube (originally mercury). It is then converted to numbers as a proxy for a patient's blood pressure level. The pressure readings are inferred to represent the pressure in blood vessels, but the movement of mercury has no direct relationship to blood pressure (Sechrest, 2005).

Temperature, described above, is often classed as an ‘objective’ measurement. Other common measures in this category include physiological indicators like heart rate, conductance, or even oxygen saturation readings from pulse oximeters. These measurements are considered ‘objective’ because they are typically obtained through standardized and quantifiable methods that are not influenced by subjective judgment or interpretation. However, like temperature these ‘objective measure’ serve as proxies for the underlying construct. As the latent variable moves from the realm of the tangible and observable to the abstract, the bridge between instrument and variable becomes more tenuous, with increased potential for error. The issues of the distance between constructs and their representative proxies are exemplified in the discipline of psychology. For example, constructs and dimensions are constantly evolving, as represented by the 70-year history of dynamic shifts in the Diagnostic and Statistical Manual of Mental Disorders (DSM). A cornerstone of Western medicine and psychiatric treatment, the DSM categories and descriptions are created through consensus and represent their time’s prevailing socio-political, ideological, empirical, and medical knowledge (North, 2015). Changes in the conceptualization of a construct or dimension inevitably result in changes to what, why, and how it is measured.

### 2.3. The consequences of flawed measurement

Unclear, poorly constructed, culturally biased, or statistically flawed instruments can lead to a multitude of negative consequences for individuals, families, and the wider community. Poor instruments may cause misdiagnoses in healthcare, with subsequent harmful interventions, suffering, and increased morbidity and mortality. This can also lead to reduced quality of life, as well as discrimination and marginalization for patients who are incorrectly labeled with conditions associated with stigma (McClimans, 2017). In the broader context, poor healthcare measurement instruments can result in policies that support or fund unhelpful treatment options. Healthcare providers and funding bodies may waste economic resources on ineffective interventions. In the research sphere, poor instruments can result in the unethical waste of resources, harmful science practices, and the dissemination of false or misleading information (Mokkink et al., 2018).

The issue of poor measurement instruments is demonstrated by Marshall et al. (2000), who examined 300 randomized controlled trials selected from the Cochrane groups register. Authors examined the instruments used in each study as published (in a peer-reviewed journal) or unpublished (i.e., scale or instrument designed by study authors or no published psychometric evidence about the scales or instruments used for data collection purposes). Randomized controlled trials were 40% more likely to claim treatment effects when using unpublished instruments. In non-pharmacological trials, one-third of claims of treatment superiority would not have been made if a published scale or instrument had been utilized. Authors hypothesized that this result might have been linked to unsound instruments, with trials that used unpublished instruments that had dropped unfavorable items (i.e., those that contradicted findings of treatment effectiveness). As the scales and instruments were unpublished, this adjustment (which contravenes good measurement practices) could occur without reviewers or editors being aware. Authors recommended that future research study manuscripts submitted for peer review should not use unpublished (and it may be inferred, unvalidated or unsound) instruments (Marshall et al., 2000). In other words, if studies submitted for review did not meet the inclusion criteria of using instruments with documented evidence of their psychometric properties, then the manuscript would be rejected before going out for peer review.

## 3. Key concepts

### 3.1. Psychometrics

Measurement is a wide and diverse field that encompasses various methods and techniques used to quantify and evaluate different attributes, properties, or characteristics of objects, events, phenomena, or concepts. It encompasses physics, engineering, economics, sociology and healthcare. In this broad field of measurement, various subfields and specialized areas exist, each dealing with specific aspects of measurement. One of these subfields is psychometrics, which is of critical significance in health care. In psychometrics, measurement properties are considered the characteristics of an instrument that describe its qualities of accuracy (validity) stability/reproducibility (reliability) and responsiveness. These qualities are therefore commonly known as the ‘psychometric properties’ of an instrument. It should be noted that validity or reliability are not immutable characteristics inherent to a measurement instrument. Instruments possess varying levels of these properties, which are influenced by the construct or factor they are measuring, the context in which they are being applied (including the target population and rater), how they are used and what the scores generated by the instrument are being used for. The latter consideration alludes to the consequence of the scores generated, with some assessments considered ‘high stakes’ (i.e., eligibility for admission to medical school or being selected as a heart transplant recipient). In contrast, others are considered ‘low stakes’ (i.e., ranking within a large group of kindergarten students on school readiness skills). Target population may influence psychometric properties; a quality-of-life instrument may demonstrate strong convergent validity with functional health status instruments in a group of oncology patients but not among patients whose primary concern is diabetes. This may be because the health-related quality of life instrument’s items were designed to link to a construct specific to oncology patients. Sechrest (2005) posits, ‘It is not measures [instruments] that are valid, but the scores that they yield and the interpretations we make of them’. This statement is perhaps an oversimplification of measurement properties, but it highlights a critical point: instrument accuracy depends on the clinician’s ability to make meaning of the scores. If the clinician administers the instrument in a non-standard manner or an untested population, scores will be affected, and validity and reliability may be severely compromised. A grasp of psychometrics is therefore an essential of good measurement and good practice. Foundational theories and terminology of psychometrics will be briefly outlined in the subsections below.

The term psychometrics arises from disciplines that were early adopters of scale and instrument creation, where decisions were based on measurement and clinical evaluation – psychology and education (Streiner, 2003). Psychometrics owes its methodological heritage to these disciplines, and is where key measurement theories such as classical test and item response theories originated (De Vet et al., 2011). Item Response Theory (IRT) and Classical Test Theory (CTT) are both methods for evaluating instrument scores and measuring patient traits. However, they differ in their approach and underlying theoretical assumptions.

#### 3.1.1. Classical test theory

CTT may be considered a ‘traditional’ approach to statistical analysis of reliability (i.e., the degree to which the measurement is free from measurement error) and validity (i.e., the degree to which the instrument truly measures what it purports to measure) (Mokkink et al., 2018). It includes well-known techniques, such as factor analysis, used to analyze numerous healthcare instruments (DeVellis, 2006; Cappelleri et al., 2014). The key concept of both CTT and IRT is that of the latent variable; the target of interest is an *unobservable* phenomenon. CTT holds that the scores produced by the instrument is a combination of the latent variable (the ‘true score’) and all the errors contributed by other influences on the observed variable (DeVellis, 2006). For example, a self-report pain instrument with a rating scale ranging from 0–10 may have errors introduced due to individual variation in pain thresholds, differences in how respondents understand the concept of pain, phrasing of the instrument items or differences in administration by clinicians. Importantly, statistical analyses utilized in CTT assume that item scores are the ‘true score’ plus *evenly* distributed random error. This assumption is linked to a notable limitation of CTT; as a result of this assumption, errors related to different items in the response scale can negate each other. This means an instrument with many items is more likely to have a balanced random error distribution. Instrument reliability can therefore be inflated by item numbers, rather than properties of individual items (i.e., the presence of highly reliable items). Redundancy (including multiple items or very similar questions in the instrument) is a foundation of precision in CTT, as it yields stronger scores on correlation analyses. The quest for item redundancy may lead the scale developer to include an excessive number of items or irrelevant items and thus incentivizes the use of semantically and syntactically uniform question structure (e.g., Do you often feel sad? Do you often feel unhappy? Do you often feel miserable?). This can undermine the accuracy and effectiveness of the instrument, as the inclusion of extraneous or overly similar items that do not truly access the underlying construct can result in a less comprehensive or accurately representative sample of the latent variable. CTT-based instruments also tend to be more sensitive to center scores (i.e., scores in the 4–6 range on a 10-point rating scale) than extremes (scores near 1 or 10 on a 10-point rating scale). Where a respondent’s score is on the extreme ends of a scale, differences in the latent variable (i.e., levels of disability, change in the quality-of-life status, or developmental change) may be poorly distinguished or outright missed. For example, a two-point drop from three to one could represent a greater degree of disability than a change from seven to five (DeVellis, 2006).

#### 3.1.2. Item response theory

A relatively modern psychometric paradigm is item response theory (IRT). Like CTT, IRT also assumes the latent variable (underlying trait) is the target of interest but approaches the measurement of the target factor or dimension differently. While CTT looks at the performance of the instrument as a whole, IRT has a ‘micro’ focus (Cappelleri et al., 2014) where it investigates each item individually, allowing for the identification of strong or weak items. This means weak items can be readily identified, revised or removed during the instrument development phase. A further advantage of item-level validation is that they remain valid, even if only a subset of items is administered. In contrast, if a CTT-based instrument is administered incompletely, the resulting score is no longer valid. The latent variable in IRT is based on interval scaling, while in CTT, it is ordinal (Kean and Reilly, 2014). Interval level measurement involves measuring a latent variable using uniform units, while ordinal level measurement involves ranking or rating the latent trait’s components. Ordinal level measurement is less precise and involves inferences; that is, intervals between values are not equal or quantifiable. When interpreting ordinal data, inferences are made based on the rank order of values, assuming higher ranks indicate greater amounts or degrees, but the actual differences between values may vary. In contrast, interval measurement is more exact and standardized. Both interval and ordinal scales can quantify the degree or amount of the latent variable present, but interval scaling provides a more detailed description.

This distinction becomes clearer when considering an analogy from horse racing, illustrated by Kean and Reilly (2014): ‘the results of a horse race can be reported as 1¼ mile times (i.e., interval), as horse lengths of victory (i.e., interval; approximately 8 feet), or as ordinal finish position listed nominally—win, place, show (i.e., ordinal), or as a numerical order—1st, 2nd, 3rd (i.e., ordinal). Ordinally scaled nominal or numerical finish position tell you which horse was best, whereas interval scales such as finish times and lengths of victory tell you how much better the winner was compared with the other horses’ Thus, a CTT-based scale can provide a relative ranking, describing which patient has the most pain. In contrast to CTT, IRT-based instruments describe how much pain patient A experiences *compared* to patient B. IRT analyses has further advantages; they can tolerate incomplete data sets, and some IRT approaches can provide rankings of individual scale items regarding respondent ability and item difficulty. However, IRT also has limitations – the large sample size required for development (up to 1,000 participants in some models) may be problematic from cost-effectiveness and feasibility perspectives (Kean and Reilly, 2014).

#### 3.1.3. Psychometrics versus clinimetrics versus measurement properties

Lastly, it must be acknowledged that some alternative terms for psychometric properties have been proposed. This includes ‘clinimetrics’ (Feinstein, 1987) which has been suggested to be more attractive to medical and healthcare clinicians who might be uncomfortable with terminology and processes inherited from psychology (Streiner, 2003; De Vet et al., 2003a). Clinimetrics has been further proposed to differ from psychometrics in some methodological issues (De Vet et al., 2003b). However, authors have also suggested that the dichotomy between the two terms is negligible (Streiner, 2003; De Vet et al., 2003a), preferring the term ‘measurement properties’ (De Vet et al., 2011). Readers investigating the field of measurement instrument development and validation should be aware that all three terms may be encountered and broadly describe the same concept; the statistical properties of an instrument that delineate its qualities pertaining to validity, reliability and responsiveness. This manuscript will use ‘psychometrics’ as the preferred terminology, in acknowledgement of the dominance of the term in the field of healthcare measurement (i.e., the search term ‘psychometric*’ resulting in >110,000 abstracts and ‘clinimetric*’ in ~1,500 PubMed free-text search results as of January 2023).

### 3.2. The COnsensus-based standards for the selection of health status Measurement INstruments

The (COnsensus-based Standards for the selection of health status Measurement INstruments) COSMIN initiative was established in 2005 in response to issues in the measurement literature – extensive unclear, imprecise, or conflicting terminology and definitions for measurement properties, gaps in evidence for many outcome measurement instruments and inconsistency of methodologies used to demonstrate evidence of measurement properties. An international consortium of scientists with specialties in epidemiology, psychometrics, medicine, qualitative research, and healthcare collaborated, intending to improve the quality of studies of measurement properties of clinical scales and instruments. The team mobilized their expertise in measurement design and validation to develop the only international, consensus-based framework for terminology, preferred analysis and reporting of measurement properties. This has led to the development of several resources for evaluating and improving the quality of instruments, including a taxonomy, risk of bias tool, study design checklist, reporting guidelines, a guideline for conducting systematic reviews, and a guideline for selecting measurement instruments for a core outcome set (Mokkink et al., 2018, 2020).

The COSMIN taxonomy categorizes measurement properties of health outcome measurement instruments into three domains: reliability, validity, and responsiveness. These are a broad category of measurement properties that the COSMIN group classifies under nine measurement properties: content validity, structural validity, internal consistency, cross-cultural validity, measurement invariance, reliability, measurement error, criterion validity (which includes criteria for sensitivity and specificity for dichotomous scores), hypotheses testing for construct validity, and responsiveness (Mokkink et al., 2018). Table 1 in the supplements provides a detailed explanation of each of these properties, along with clinical examples to illustrate their application. This table is a conceptual guide to the COSMIN measurement properties and their statistical analysis. Table 2 in the supplements shows how various terms used in psychometric literature are mapped to the COSMIN terminology.

**Table 2:** Instrument development and review practices applied examples from the VMV (Swan et al., 2022).

	VMV example
Instrument review	
Step one: Identify existing instruments in a predefined content area and population Establish the current state of practice in the field Collate existing instruments	Developers systematically searched databases for published manuscripts describing the psychometric properties of visuoperceptual measurement instruments for both VFSS and FEES recordings. These processes are complementary ‘gold-standards’ and instruments for both accessing similar constructs and using similar items.
Step two: Retrieve data on psychometrics for existing instruments	Developers reviewed the psychometric properties of existing instruments for VFSS and FEES. Evidence of psychometric properties was extracted from manuscripts describing the instruments’ development, validation or use.
Step three: Compare retrieved data with pre-defined criteria Determine the quality studies describing psychometric properties of the existing instruments Determine the quality of psychometric properties Determine if the instruments target the desired construct	Psychometric data were assessed against quality criteria from the COSMIN group. The quality of each property was then rated: Indeterminate, Not Evaluated, Conflicting, Limited, Moderate or Strong. Although 39 instruments were identified, none displayed satisfactory properties across COSMIN domains, indicating the need to develop a novel instrument.
Instrument development	
Step four: Determine the construct Define the construct to be measured Define the target population and stakeholders Define the purpose of the instrument	Developers considered the scope of the instrument (adult oropharyngeal dysphagia, but excluding esophageal swallowing), end-users (allied health and physicians, clinicians and researchers), the target population (adults with dysphagia, but excluding radically altered head and neck anatomy to support homogeneity of the sample) and the purpose (purely visuoperceptual; excluding specialized software or automated measurement instruments). Decisions were informed by literature and driven by knowledge of current clinical practices and the scope of practice of various professions.
Step five: Generate the item pool Deductive – select and modify items from existing instruments Inductive – develop items from observations/stakeholders Develop two-five times the desired final number of items	Aspects of the target construct and items assessing each were identified from the literature review. An e-Delphi (an online, anonymous survey of multiple rounds that establishes consensus on a topic) was conducted to canvass experts for opinions on the aspects to assess and how they should be assessed. Experts were presented with definitions of assessable aspects of VFSS from the literature and asked to rate their agreement with each statement or suggest modifications. They were then asked to consider several options for operationalizing (defining and measuring a construct clearly and consistently) each aspect. Combining existing literature (deductive approach) with expert opinion (representing the most recent thinking in the area) establishes sound content validity.
Step six: Develop response scales Identify the type of item (e.g., continuous, categorical) and match the response scale type Consider the number of steps required to capture the continuum of the variable – restrict to less than seven	Developers categorized each aspect identified from the literature and the Delphi study as either spatial, anatomical, temporal, or patient response (e.g., cough). Response scales were developed for each item, grounded in the Delphi’s results.
Step seven: Expert review of the draft instrument Recruit experts that represent multiple stakeholder groups Conduct cognitive interviews	Experts from disciplines of otorhinolaryngology, radiology and speech language pathology and experts with specialized training in psychometrics reviewed the first draft of the instrument together over several days. The draft was tested in a single patient over these days, with each expert providing (a) their rating and (b) verbalizing their rationale for why they selected that rating. Questions and disagreements were discussed until consensus was reached and the draft modified accordingly. This method establishes face validity; each item must appear acceptable and relevant.
Step eight: Pilot the instrument Trial instrument in a small sample of the target population User feedback; item comprehensibility, comprehensiveness, relevance, instrument acceptability and feasibility	A sample of 40 patients was collected using a standardized administration protocol and VFSS rated with the draft VMV. Raters were multi-disciplinary and kept a log of questions/issues encountered as they used the draft VMV to inform the examination of implementation experience (i.e., feasibility).
Step nine: Item reduction and revision Statistical analysis User feedback Identify poorly fitting, unsuitable or unstable items and remove or modify	The VMV was constructed using a reflective model, where items are aspects of the underlying construct (refer to Figure 3 for an example). Therefore, item reduction involved both statistical analysis (factor analysis) and user feedback from multidisciplinary experts who used the instrument in the pilot study. Items that were unstable, non-feasible, less clinically relevant or evidenced floor/ceiling effects were removed or modified.
Step ten: Trial the instrument Trial revised instrument in a large sample Repeat statistical analyses to determine the psychometric properties of the final version of the instrument	Currently ongoing, this trial involves multiple sites in multiple countries, with a target of 300 participants. The final statistical analysis will include both classical test theory factor analysis and item response theory analyses.

Since its inception, COSMIN tools and taxonomy use has experienced substantial growth. The COSMIN group maintains a database of systematic reviews of studies of measurement properties of instruments in healthcare. Although the COSMIN framework was developed specifically for evaluating Patient-Reported Outcome Measures (PROMs), the framework’s principles can be adapted and applied to other types of measurement instruments, including clinical observation instruments (Mokkink et al., 2018). To date, over 350 of the systematic reviews utilized COSMIN terminology or tools.^1^ The COSMIN taxonomy and tools represent international, research-based practice in measurement and statistics for instruments in healthcare. Therefore, this manuscript will consider measurement properties and instrument development through the lens of the COSMIN.

### 3.3. Describing and understanding the properties of a measurement instrument

Instrument development necessitates the integration of a robust theoretical framework, sound methodological protocols and appropriate statistical analyses. The results of these analyses describe the properties of an instrument. Like any field of science, there are a variety of methodologies with varying strengths and weaknesses in different statistical approaches. The COSMIN group recommends preferred statistical analyses, sound study design and criteria for good measurement properties (Mokkink et al., 2020). Table 1 lists preferred analysis methods for the psychometric properties recommended by the COSMIN group and briefly introduces the interpretation of these analyses. This table provides an accessible, non-mathematical overview of the suggested statistical analyses, focusing on concepts rather than procedural knowledge. It is designed to support non-statisticians at an introductory level.

## 4. Instrument development

**Figure 2:**
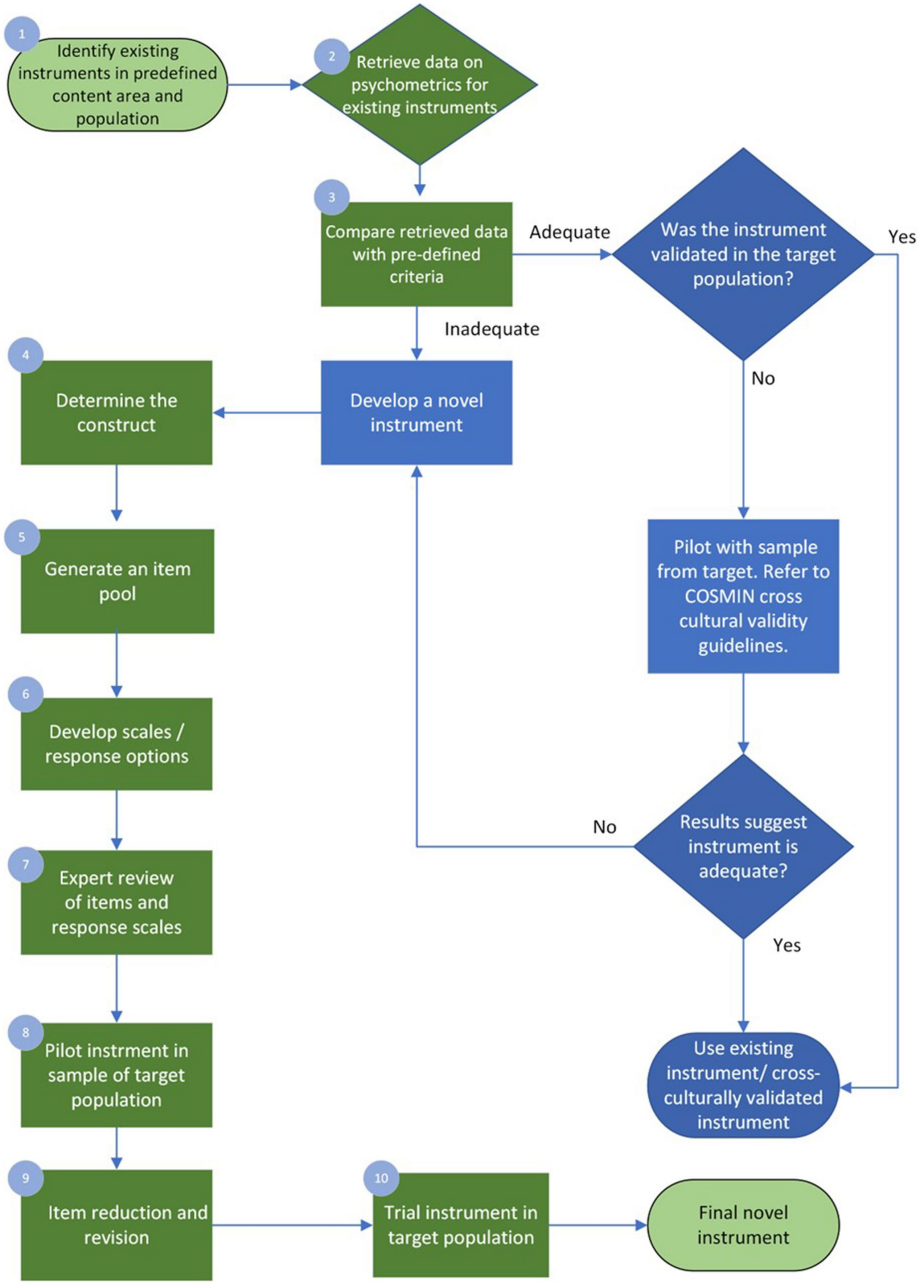
10-step Process of Instrument Development.

When the clinician, academic educator, or researcher finds the available instruments are inadequate regarding psychometric properties or feasibility or a poor match to their target population or question, they may be interested in developing a new one. The following section illustrates one possible path for instrument development. Examples are drawn from the visuoperceptual measure for videofluoroscopic swallow studies (VMV), a recently developed instrument for the analysis of videofluoroscopic studies of dysphagia, dynamic x-rays of difficulty swallowing, developed in adherence with COSMIN guidelines and summarized in Table 2 of this manuscript. The ten-step process discussed below briefly demonstrates COSMIN guidelines in the applied practice of instrument development, with the process summarized in Figure 2.

### 4.1. Step one: identify existing instruments in a predefined content area and population

As with all scientific endeavors, a thorough understanding of existing work and gaps in the respective field is required initially. A detailed and rigorous examination of the existing instruments will result in common themes and identify gaps and future directions. Reviewing the related empirical research literature should be critical and systematic (Streiner et al., 2015; Page et al., 2021). The VMV began with a psychometric review, wherein all visuoperceptual measurement instruments (those which rely on human visual inspection and judgment of images) for ‘gold-standard’ technical assessments of swallowing were collated, and their measurement properties critically examined according to COSMIN quality criteria (Swan et al., 2019). In this context, the term “gold standard” refers to the highest achievable standard backed by evidence and expert agreement (Ralph et al., 2015). The psychometric evaluation therefore focused on instruments used in the analysis of Videofluoroscopic Swallow Studies (VFSS) and Fiberoptic Endoscopic Examinations of Swallowing (FEES) as there is currently no agreement on which measure of VFSS or FEES can be considered a gold standard (Giraldo-Cadavid et al., 2017). The COSMIN initiative specifies detailed recommendations for conducting a systematic review of measurement properties.

### 4.2. Step two: retrieve data on psychometrics for existing instruments

Experts in health-scale measurement and psychometrics agree that novel instrument development should only be undertaken if absolutely necessary (De Vet et al., 2011; Streiner et al., 2015). Quality instrument development is a time-and resource-intensive process that may take months or years. Clinicians, academic educators, and researchers should consider whether a novel instrument is truly required. This decision should be informed by both the results of step one and their intended purpose for the new instrument. The psychometric review that led to the development of the VMV identified 39 instruments across 45 studies (Swan et al., 2019). Ideally, step one will identify one or more instruments with sound psychometric properties that meet the needs of the clinician, educator or researcher and are validated for use in the target population. However, before selecting an instrument, the healthcare professional must evaluate the quality of existing instruments to determine which is best suited for their intended purposes. This includes considering the instruments’ psychometric properties, as well as their validation in the specific target population of interest.

### 4.3. Step three: compare retrieved psychometric data with pre-defined criteria

The psychometric data retrieved in the review must be assessed with quality criteria defined *a-priori*. The COSMIN group generated specific quality criteria per each aspect within domains of validity and reliability and interpretability (Mokkink et al., 2018). For example, 75% of formulated hypotheses for construct validity should be confirmed to meet the requirements for good measurement properties. Hypotheses can refer to the expectation of no significant changes between genders or age groups where age or gender would not mediate the disease. Uld not mediate the disease. The COSMIN initiative provides detailed guidance for this process [7] An instrument may be used without modification if it demonstrates adequate psychometric properties and is both suitable and feasible for the intended target participant group. Often this is not the case, and the measurement instrument requires modification to meet the intended purpose (such as trialing in the target population). If an instrument can be modified to suit the intended purpose, it will require testing before its reliability, validity, and responsiveness can be assumed. Psychometric properties are ‘non-transferrable’ – modification to the instrument or use in an untested population may introduce unknown variables that affect instrument performance and accuracy. The COSMIN guidelines provide instructions on assessing the cross-cultural validity of an instrument and should be applied in this situation (Mokkink et al., 2018).

Alternatively, the clinician, educator or researcher may conclude that instrument development is required. Where there is a need for existing instruments in the construct of interest and target population (e.g., age, diagnosis), unsound psychometrics and poor feasibility of existing measures, the development of the needed instrument should progress. In the case of the VMV’s psychometric review, none of the existing 39 measures demonstrated adequate psychometric properties to recommend them for clinical practice or use in research (Swan et al., 2019). It is beyond the scope of this paper to elaborate on specific methodology for psychometric review and quality analysis; interested readers should refer to COSMIN texts (De Vet et al., 2011; Mokkink et al., 2018) and may refer to Swan et al. (2019) for an example of the review and quality analysis.

### 4.4. Step four: determine the construct(s)

To develop the VMV, the construct of interest required a formal definition. The construct is the ‘well-defined and precisely demarcated subject of measurement’ (De Vet et al., 2011). It should be defined *a priori* and specific to the instrument’s intended function. The importance of a well-described construct is particularly evident in the context of an unobservable target construct, a phenomenon common to health-related constructs. It is critical to be precise and unambiguous on what is to be measured and how it differs from other constructs (DeVellis and Thorpe, 2021) as non-specific instruments risk missed or inappropriate diagnoses, inaccurate treatment monitoring or unclear outcome measurement instruments. If a construct is multidimensional, the instrument developer must be clear on the aspect(s) to be measured – for example, anxiety contains multiple aspects linked to personality, stimulus, and contextual situation. The target population (patients and the stakeholders who will use or interpret the instrument) and the purpose of measurement should also be considered at this stage (De Vet et al., 2011). Health measurement instruments may broadly be classified as diagnostic, prognostic or evaluative (McDowell, 2006).

Diagnostic instruments refer to devices or measurement tools that aid in clinical diagnosis. Some examples include sphygmomanometers for hypertension, mental health self-report surveys for anxiety or in this case and VFSS for oropharyngeal dysphagia, and. Prognostic measurement instruments include screening tests like Apgar scores (Apgar, 1952) and the Geriatric Depression Scale (Janneke et al., 2012); these measure future potential of function or dysfunction. Evaluative instruments track change over time. More discrete divisions of the scope of the instrument are also possible, such as discriminative (classifying individuals based on IQ) or instruments concerned with a particular aspect of health, such as quality of life (McDowell, 2006). Clinicians/educators/researchers engaged in instrument development must match the purpose of the instrument to the appropriate instrument design. In the case of the VMV, literature and expert consultation was used to determine the scope and design of the instrument (i.e., excluding esophageal dysphagia from the construct of ‘disordered swallowing’ for the purposes of this measure, creating a measure that was visuoperceptual to reflect both current practices and support future ease of use and access for sites unable to purchase specialty software).

### 4.5. Step five: generate the item pool

The item pool can be developed after clearly delineating the construct and related aspect(s). At this stage, having clear specifications or operational guidelines for creating the “item pool” is essential because it helps prevent potential bias. Without such guidelines, there’s a risk of disproportionately emphasizing certain indicators over others during the initial phase. This imbalance in measurement can subsequently influence the definition of the construct, leading to an inaccurate representation of the intended concept. There are multiple possible methods, including the ‘deductive’ and ‘inductive’ approaches. In the deductive method, scale item generation results from completing a literature review and collecting and selecting items from previously designed instruments (Boateng et al., 2018). Publicly available item banks of instruments with known psychometric properties, such as the Patient-Reported Outcomes Measurement Information System (PROMIS) and the library of clinical outcome assessments,^2^ may assist the clinician, educator or researcher at this stage. In contrast, the inductive method relies on key stakeholders’ input to generate items, which may include focus groups, surveys, or observational/exploratory research develops items (Boateng et al., 2018). Focus groups may involve a small group of stakeholders impacted by the target disorder interviewed by a facilitator, with answers recorded, transcribed and analyzed. Care must be taken with selection of the group members with regards to hetero or homogeneity, depending on the aims of the interview. Key informant interviews involve more detailed interviews with a small number of crucial participants with deep subject knowledge. Interviews typically continue until saturation is reached – i.e. interviewing additional informants does not add new knowledge. Qualitative approaches such as these may be an excellent source for developing items assessing subjective symptoms (Streiner et al., 2015). It should be noted that crowd-sourcing of items requires instrument developers to have the construct well-defined to develop well-worded questions that elicit useful responses (De Vet et al., 2011). Combining both methods will likely be most efficacious in most cases, as the literature review provides a sound theoretical basis, while iterative, qualitative data collection generates items closely aligned to the intended population or purpose (Boateng et al., 2018).

Items should be worded with great care. The instrument’s quality largely depends on the quality of its items; that is, it’s content (Streiner et al., 2015). Content validity describes the correspondence of the contents of the instrument to the target construct and is established through relevance and comprehensiveness of the instrument’s items (De Vet et al., 2011). Assessment of content validity is further outlined in Table 1. The COSMIN initiative provides detailed instructions for reviewing content validity (Mokkink et al., 2018). Item phrasing and language should therefore be specific, unambiguous, constrained to one concept per item, avoid negative wording, and be at an appropriate reading level, especially in consideration of the target population’s general level of education and health literacy (De Vet et al., 2011). At this stage in instrument development, the clinician, educator or researcher should aim for the similarity between items. This similarity is termed ‘redundancy’ and is a desirable feature in the early stages of scale item generation (Streiner et al., 2015). As the items are intended to measure the same construct, multiple similar items support good content coverage – that is, a comprehensive and representative sample of the components and aspects that make up the target construct are included (DeVellis and Thorpe, 2021). The consequence of redundancy is that the initial item pool will be considerably larger than the intended end product; authors have suggested that the initial item pool may be two to five times greater than that of the final version of the instrument (Boateng et al., 2018). The VMV’s initial pool included 97 items, developed through both a deductive and inductive approach; that is, literature review, review of existing instruments, consultation with key stakeholders and crowd-sourcing items through the e-Delphi approach (Swan et al., 2022). Step nine of the instrument development details removal and refinement of the items that do not meet pre-defined criteria for internal consistency (excessively or insufficiently high Cronbach’s Alpha).

### 4.6. Step six: develop response scales

A response scale is the orderly quantification of the item (an aspect of the construct under assessment). Broadly speaking, response scales may be *categorical* (religion, marital status, eye color) or *continuous* (mass, pain rated on a 100 mm line, temperature). More specifically, in the categorical division, scales could be *nominal* (two response categories; e.g., hot or cold, inside or outside, urban or rural) or *ordinal* (categories with natural ordering; e.g., very unhappy, unhappy, neutral feeling, happy, very happy). If the distance between each step in the scale is equal, the scale is called *interval.* The variable being measured must match the response scale. For example, an interval scale could measure body temperature in degrees Celsius or be rated as very low, low, within the expected range, high, or very high (Streiner et al., 2015). The type of response scale used informs the response options’ design, formatting, and wording. Certain items may only be suitable for specific types of response scales. A dichotomous scale must have unambiguous language, as there is no middle ground. In contrast, a Likert-type scale must ensure that both extremes of the variable (e.g., strong presence vs. total absence) are clearly captured at the start or end of the scale (Boateng et al., 2018). The steps in the response scale should be presented in a logical order (least to most), and the number of points on the scale should be limited to seven or fewer. The human capacity to process distinctions in categories becomes an unwieldy cognitive load above this number (Miller, 1956). The type of response scale should also consider the target respondent group. For example, response scales used with children would need to consider their developmental level and response scales used with respondents with diverse cultural backgrounds would need to ensure that the wording of items is culture and bias free.

The items of the VMV were developed using the e-Delphi process. The constructs to be assessed were operationalized into a measurable format, such as the fraction of a bolus, the anatomic space filled, or the temporal or spatial movement of a swallowing structure. These were then refined into ordinal scales with less than seven response options that corresponded to the degree of presence or absence of the constructs.

### 4.7. Step seven: expert review of the draft instrument

A group of experts should review the new draft instrument. Ideally, this should include individuals or groups who are the target end users of the instrument and highly experienced in psychometrics. The group should assess the instrument’s face validity and refine the measurement instrument before pilot testing it. Adequate face validity indicates that the instrument fits the target construct (Boateng et al., 2018). Cognitive interviews are recommended when the unconscious mental processes of completing the instrument or interpreting its items are explicitly verbalized (De Vet et al., 2011; Boateng et al., 2018). The VMV utilized a multi-disciplinary group of experts to provide feedback on aspects of its items in testing the measure across multiple patients (Swan et al., 2022).

### 4.8. Step eight: pilot testing the instrument

The next step in instrument development is the pilot testing process. The first draft should be tested in a small sample of the target population (24–36 respondents are suggested for this phase) (Johanson and Brooks, 2010) and then refined with due consideration of the psychometric performance and user feedback on the instrument’s implementation and interaction experience. Important concepts to consider in user analysis of the pilot version of the instrument are item comprehensibility, relevance, instrument acceptability, feasibility, the cognitive load required to answer items, time taken to complete items, and comprehensiveness. Acceptability is whether participants are willing to undertake the measurement test or activity, while feasibility denotes whether they have the ability to do so (De Vet et al., 2011). The VMV was piloted with 40 patients, and the measurement instrument tested by a speech-language pathologist two physicians with specialties in radiology and ENT-phoniatrics (Swan et al., 2022).

### 4.9. Step nine: item reduction and revision

After the pilot testing phase and the results are analyzed, item reduction and revision may occur. Reduction and revision involve removing or modifying items that were unsuitable from user experience perspectives or quality of psychometric properties. Items may be deleted outright, moved to another section of the instrument, collapsed with another item (or more) for a single item, or (more rarely) split to make two novel items. Rating scales are also subject to modification, with removal, rewording or collapse of steps. Item reduction and revision ensure that only the items with the strongest relationship to the construct remain (Boateng et al., 2018). The VMVs items were revised through cognitive interviewing with the raters and statistical analysis to remove or modify weak items (Swan et al., 2022).

**Figure 3:**
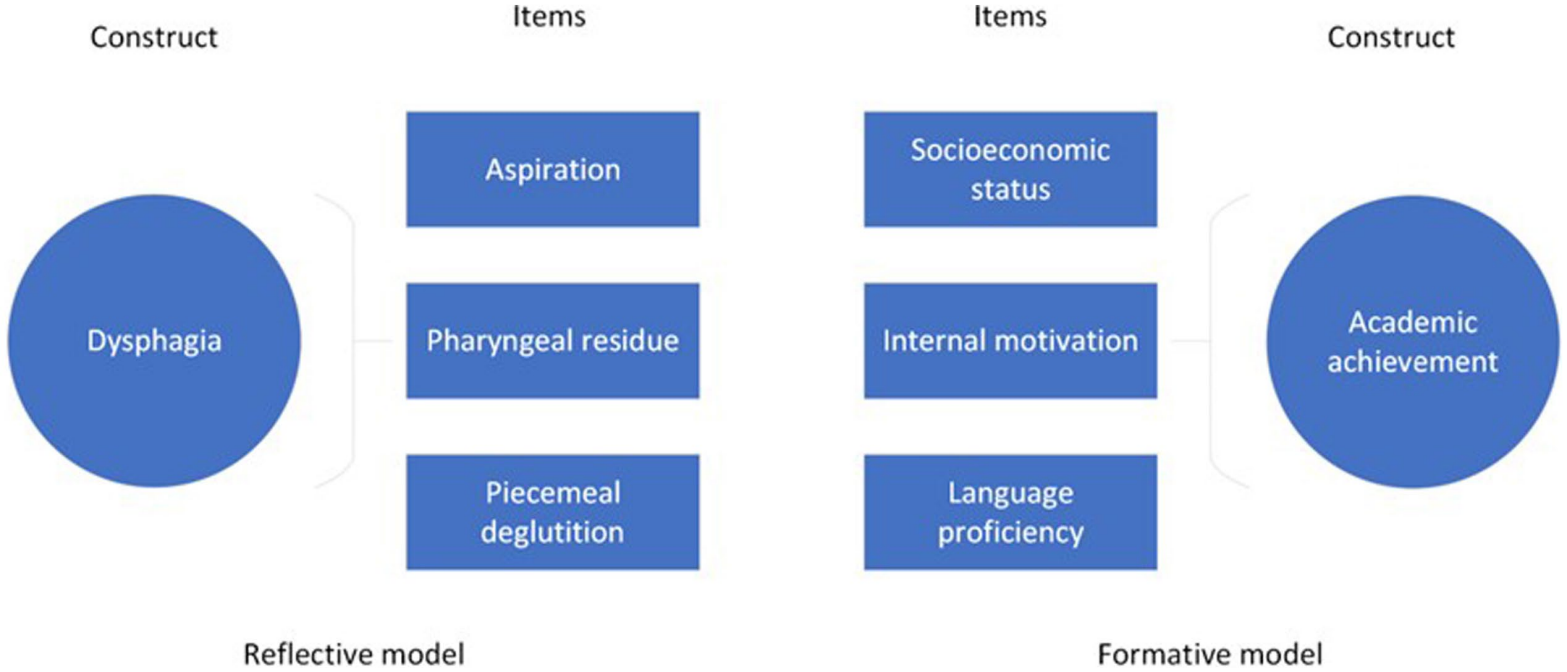
Reflective and Formative Models.

The underlying theoretical underpinnings inform item reduction and revision processes of the instrument; that is, whether it was formed using a reflective or formative model (i.e., where the items are assumed to be accessing a single underlying construct or trait versus an instrument that is tapping into multiple sub-constructs or components that make up target construct). Figure 3 demonstrates an applied example of these models, where the items ‘aspiration’, ‘pharyngeal residue’ and ‘piecemeal deglutition’ arise from the single construct ‘dysphagia’ compared to the construct ‘academic achievement, which is comprised of sub-constructs ‘socioeconomic status’, ‘internal motivation’ and ‘language proficiency’.

In the reflective model, factor analysis and item correlation guide both item reduction and identification of viable dimensions. Patient judgment of the importance of items guides retention or rejection of items in instruments constructed with the formative model, such as self-report surveys (De Vet et al., 2011).

### 4.10. Step ten: trialing the instrument

The natural consequence of item reduction and revision is creation of an instrument that is substantially different to the version that was piloted. The VMV was reduced in length by ~50% between the pilot and trial phases (Swan et al., 2022). The new, reduced version of the instrument requires testing and evaluation of psychometric quality in a larger sample of the target population (De Vet et al., 2011). This version of the instrument will be penultimate; developers can expect to require further revisions after trial and psychometric analysis. At this stage, statistical analyses (e.g., factor analysis, LOA) are appropriate; however, these may require a large sample (in the hundreds, in the case of IRT) to meet their statistical assumptions (Kean and Reilly, 2014). A final round of item removal and edits will ensue, with repeated statistical analyses to judge the impact of individual items and response scales on instrument psychometric properties. Once a satisfactory instrument structure has been attained, the final version is ready for clinical use.

## 5. Discussion

This paper introduced key concepts in psychometrics for healthcare clinicians, academic educators, and novice researchers interested in measurement and outlined the process of best-practice instrument development and validation in a practical, accessible, manner. This paper used the recently developed VMV (29) as an exemplar of one possible pathway for instrument development that adheres to the COSMIN initiative recommendations and guidelines.

This manuscript has discussed the complexities inherent in measurement in healthcare, the *ballung* concepts and latent constructs. It has outlined that while validity and reliability constitute important measurement instrument quality domains, validity is the most critical domain. Reliability is moot if an instrument has strong reliability but items that do not adequately represent the latent target variable, regardless of whether the instrument is repeatable or consistent. In other words, validity trumps reliability in the psychometric property hierarchy in instrument development (Lissitz, 2009). An instrument that is reliable but does not have evidence of validity is useless, precisely because of the issues arising from *ballung* and latent constructs. In most cases, the target construct under investigation in healthcare is often high-stakes, involving a life-threatening disease, expensive long-term management, and significantly impacting quality of life. Therefore, adequate evidence is required before a measurement instrument can be trusted. Psychometrics can provide that empirical evidence that underpins sound measurement (Vetter and Cubbin, 2019). The COSMIN initiative is a resource that can guide clinicians and researchers interested in addressing instrument quality and development.

### 5.1. Poor measurement in healthcare – a widespread issue

Unfortunately, poor measurement in healthcare is widespread. In 2011, authors (Cano and Hobart, 2011) noted the lack of quality psychometrics of instruments used in healthcare settings, evident in even the most cursory review of the peer-reviewed literature. The authors gave the example of a superficial literature review of randomized controlled Phase III and IV trials in multiple sclerosis published in 2006–2011. Fourteen of 28 articles used some type of rating scale, but only two studies used scales with any published psychometric evidence. Over a decade later, the situation remains essentially unchanged. In a review of 80 outcome measures used for 100 randomized controlled trials in narcolepsy interventions, authors found ‘surprisingly little evidence for the validity, reliability, and responsiveness of [the instrument] frequently used to assess treatment efficacy in narcolepsy’ (Schokman et al., 2022). Unfortunately, this pattern is repeated in various disciplines, across different populations and diagnostic categories. In numerous reviews, authors consistently find that the psychometric evidence for instruments is poor, limited, or lacking in quality (Crellin et al., 2015; Cassidy et al., 2018; Ghai et al., 2022; Thompson et al., 2023).

The dearth of quality instruments in healthcare clearly indicates to the clinicians, educators, and researchers that it is in the patients’ and health professionals’ best interests to engage with psychometrics and upskill in knowledge in the field. Too often, instruments are developed poorly, leading to all subsequent data and treatment findings based on their data being questionable (Schokman et al., 2022). Instrument development is a dynamic, iterative process involving creativity, theory, statistical evaluation, and dedication from the instrument authors. Instrument development should not cease at the first draft; rigorous field testing using best-practices and appropriate statistical analysis is required (De Vet et al., 2011).

### 5.2. Implications for clinicians, educators, and researchers

- Measurements matter. Healthcare professionals should understand the importance of well-designed and feasible measurement instruments with robust psychometric properties in their work. These instruments play a crucial role in evidence-based practice and good research, as they provide a way to assess the populations, interventions, or other instruments accurately.
- The COSMIN (COnsensus-based Standards for the selection of health Measurement INstruments) initiative provides a wealth of materials and guidelines to support researchers and clinicians in selecting and using psychometric instruments. COSMIN resources can assist clinicians in developing, researching, and selecting measurement instruments with robust psychometric properties that are appropriate for their intended use.
- Psychometrics apply to healthcare across all disciplines, from mental health to internal medicine to rehabilitation science.
- The development and validation of the VMV is one example of applied COSMIN initiative recommendations and may serve as a useful guide for clinicians and researchers interested in instrument development or validation.
- This manuscript calls for a concerted effort among clinicians interested in psychometrics research, serving as a rallying point by presenting key concepts and principles and providing guidance for their practical application in instrument development and selection.

## Author contributions

KS, RS, TB, and RC contributed to conception and design of the review. MS, DF, and VW provided data used in the VMV development and contributed to validation, the processes of which are described in this manuscript. KS and RS: original draft preparation. TB and RC: review and editing. All authors have read and agreed to the published version of the manuscript.

## Funding

The authors wish to acknowledge Curtin University and the Australian Federal Government for the Curtin University Postgraduate Scholarship (CUPS) and the Australian Postgraduate Award (APA).

## Conflict of interest

The authors declare that the research was conducted in the absence of any commercial or financial relationships that could be construed as a potential conflict of interest.

## Publisher’s note

All claims expressed in this article are solely those of the authors and do not necessarily represent those of their affiliated organizations, or those of the publisher, the editors and the reviewers. Any product that may be evaluated in this article, or claim that may be made by its manufacturer, is not guaranteed or endorsed by the publisher.

## Footnotes

^1^ www.cosmin.nl/database/

^2^ www.mapi-trust.org
