# Supplementary material for: Measuring what matters in healthcare: a practical guide to psychometric principles and instrument development
Source: Front Psychol. 2023 Sep 18;14:1225850. doi: 10.3389/fpsyg.2023.1225850 (PMC10543275; doi:10.3389/fpsyg.2023.1225850)
Supplement: Supplementary file 1 [file Data_Sheet_1.docx]

Supplementary Material

**Measuring What Matters in Healthcare: A Practical Guide to Psychometric Principles and Instrument Development**

**K., Swan1,2,3 ORCiD: 0000-0003-4530-5548, R., Speyer1,4,5* ORCiD: 0000-0003-2828-8897, M., Scharitzer6 ORCiD: 0000-0003-4427-5463, D., Farneti7 ORCiD: 0000-0002-8139-4990, T., Brown8 ORCiD: 0000-0001-9403-5877, Woisard, V ORCiD, R., Cordier1,10,11 ORCiD: 0000-0002-9906-5300* Correspondence:** Renée Speyer: renee.speijer@isp.uio.no


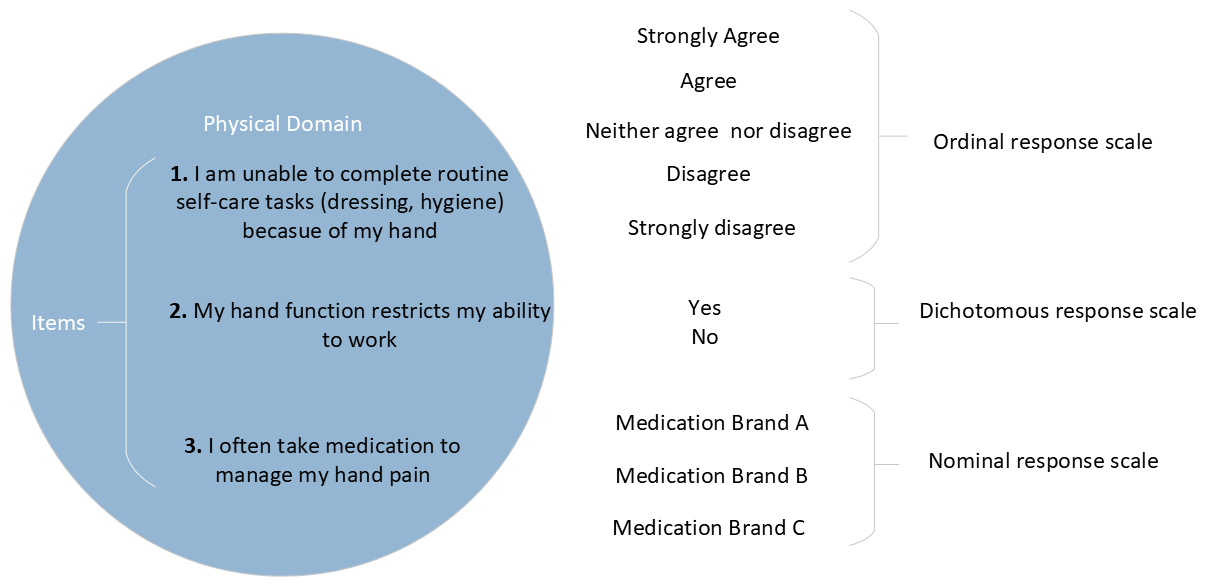


**Supplementary Figure 1.** Example of components of a measure

Measurement instruments may focus on a single aspect (e.g., thumb range of motion post-hand surgery) or wider domains (e.g., physical domain of impact hand injury). The items can be operationalised in different ways (e.g. define and quantify hand pain by the amount or type of daily medication required) and will have response scales to match the operationalisation.

**Supplementary Table 1:** COSMIN terms and definitions (Mokkink et al., 2018), modified with extended examples

| **Aspects of Psychometric Properties** |  | **Definitions^a^** | **Examples** |
| --- | --- | --- | --- |
|  | **Domain** | **Validity**  **The degree to which an instrument measures the construct(s) that it purports to measure** | *Does the instrument really measure what it claims to? How well does it function as a proxy for the construct?* |
|  | **Content Validity** | The degree to which the content of an instrument is an adequate reflection of the construct being measured (includes face validity) | *An instrument should cover the construct of interest comprehensively and comprehensively. That is, everything that is relevant to the construct is included in the instrument* *and makes conceptual sense. For example, a measurement instrument of cognition that did not include tasks targeting working memory would not be comprehensive. If the instructions for administration or scoring were unclear, it would not be comprehensible. If the same instrument* *included items about hair colour, it would not be relevant.* |
|  | Face validity^b^ | The degree to which instrument (items) indeed looks as though they are an adequate reflection of the construct being measured | *The instrument* *includes items and is constructed in a way that would accurately measure the target. A clinician would recognise the items as being relevant to the target – for example, a measurement instrument of reading comprehension post-stroke that included items sampling many different types of writing would have good face validity. The face validity would not be supported if the same instrument* *included numeracy and drawing tasks and purported its scores to pertain to reading solely.* |
|  | **Construct Validity** | The degree to which the scores of an instrument are consistent with hypotheses *(for instance, concerning internal relationships, relationships to scores of other instruments, or differences between relevant groups)* based on the assumption that the instrument validly measures the construct to be measured | *Assuming the instrument* *truly measures the construct of interest, how do its scores behave under conditions where it is hypothesised they may be impacted? For example, two instruments* *of sitting balance would be expected to be related – if the scores on one decreased, it is expected the other would decrease if the hypothesised relationship is true. Suppose an instrument* *of potential to regain independent walking ability with a prosthesis post-amputation is accurate. In that case, it is hypothesised it would show different scores when tested in patients who did walk independently and those who never have.* |
|  | Structural validity | The degree to which instrument scores are an adequate reflection or representation of the dimensionality of the construct or factor being measured | *Dimensionality describes if the target construct has multiple facets (multidimensional) or a single dimension (unidimensional). This can be statistically tested, and the scores the instrument* *attains in this instance should reflect the hypothesised nature of the underlying construct/dimension. A complex construct, like anxiety, would be expected to be multidimensional. Therefore, an instrument* *for anxiety should likewise be multidimensional to access all relevant components of the construct. A simple construct, like plasma albumin level, would be unidimensional, and the instrument* *should reflect this.* |
|  | Hypothesis testing | Idem construct validity |  |
|  | Cross cultural validity | The degree to which the performance of items on a translated or culturally adapted scale or instrument adequately reflects the performance of the items in the original version of the scale or instrument. | *Cross cultural validity is not restricted to instruments translated from one language to another. It reflects the correlation between the original measure and a measure adapted from the original to a different population; this may be a different cultural group, patient cohort or speakers of another language.* |
|  | **Criterion Validity** | The degree to which the scores of a measurement instrument are an adequate reflection of another instrument or scale deemed a ‘gold standard’. | *Gold-standard in measurement science is a benchmark that is the best available, under reasonable conditions. It is not the perfect test, but merely the best available standard with known results. An instrument with good criterion validity will strongly correlate with the ‘gold standard’ scores for the target construct.* |
|  | **Domain** | **Reliability**  **The degree to which the measurement instrument is free from measurement error** | *Is the instrument* *free of errors? Will it remain stable if repeated over time, in different contexts / by different raters? Do the relationships between items make sense?* |
|  | **Internal Consistency** | The degree of correlation among the items of a score or instrument. | *How the items in the instrument* *fit together – a scale about depression with items about preferences when buying shoes would represent two different constructs and have poor internal consistency until those items are removed. Low internal consistency may indicate an item in the instrument that does not belong there or excessive redundancy of items (high cross-over among items).* |
|  | **Reliability** | The proportion of the total variance in the measurements which is due to “true” differences between patients  May be further divided into: Intra-rater reliability, inter-rater reliability and test-retest reliability; agreement within the same rater, between raters, and consistency of scores at different times respectively. | *A hallmark of statistical theory in measurement (classic test theory) is the assumption that error is present in measurement. Therefore, the ‘true’ score is impossible to access – the scores the instrument* *produces are the summation of the ‘true’ score and random errors surrounding the score. Statistical analysis of this property attempts to measure if the magnitude of difference in scores between patients/clinicians is because the ‘true’ scores are different or because the other errors affected one or both scores.* |
|  | **Measurement Error** | The systematic and random error of a patient’s score that is not attributed to the true changes in the construct to be measured. | *All instruments* *are vulnerable to error; this may be inherent in the design (poorly worded items), administration errors or interpretation mistakes. Statistical analysis of the properties of instruments* *assumes and accounts for some level of random error in the ‘true score’ the instrument* *produces. Therefore, ideally, this score would be low.* |
|  | **Domain** | **Responsiveness**  **The ability of an instrument to detect change over time in the construct to be measured** | *The way the instrument* *tracks change over time; for example, the instrument* *accurately reflects the improvement in patients' lung function pre and post a therapeutic trial.* |
|  | **Responsiveness** | Idem responsiveness |  |
|  | **Domain** | **Interpretability^d^**  **The degree to which one can assign qualitative meaning – that is, clinical or commonly understood connotations – to an instrument’s quantitative scores or change in scores.** | *Although not considered a psychometric property, interpretability is important as it influences the ‘useability’ of the instrument. The instrument's* *scores translate to clinical significance or impact; for example, a measurement instrument that provides a single score for the patients' orientation that is meaningful for clinicians and accurately represents a change from delirium to oriented would have strong interpretability.* |

*Notes*

aApplies to Health-Related Patient-Reported Outcomes (HR-PRO) instruments.

bAspect of content validity under the domain of validity.

cAspects of construct validity under the domain of validity.

dInterpretability is not considered a psychometric property

**Supplementary Table 2:** COSMIN terms (Mokkink et al., 2018) matched to similar terms (Denman et al., 2017). Reproduced with permission from the publisher with additional terms included.

| **Domain** | **COSMIN Psychometric property and definitions** | **Examples of terms used outside of COSMIN that may relate to measurement property** |
| --- | --- | --- |
| **Reliability** | **Internal consistency** (The degree of the interrelatedness between items)  **Reliability** (Variance in measurement instruments which is because of ‘true’ differences among clients)  **Measurement error** (Systematic and random error of a client’s score that is not due to true changes in the construct to be measured) | Internal reliability  Content sampling  Conventional item analysis  Inter-rater reliability  Inter-scorer reliability  Intra-rater reliability  Test-retest reliability  Temporal stability  Time-sampling  Parallel forms reliability  Split-half reliability  Alternate form reliability  Standard Error of Measurement |
| **Validity** | **Content Validity** (the degree to which the content of an instrument is an adequate reflection of the construct or factor being measured)  **Construct Validity** (The degree to which scores are consistent with hypotheses based on the assumption that the instrument validly measures the construct being measured)  **An aspect of construct validity – structural validity** (The degree to which scores reflect the dimensionality of the measured construct)  **An aspect of construct validity – hypothesis testing** (item construct validity) | n/a  n/a  Internal structure  Concurrent validity  Convergent validity  Divergent validity  Know group validity  Predictive validity  Discriminant validity  Contrasted groups validity  Identification accuracy  Diagnostic accuracy |
|  | **Aspect of construct validity – Cross-cultural validity** (The degree to which the performance of the items on a translated or culturally adapted instrument are an adequate reflection of the performance of the items of the original version of the instrument)  **Criterion validity** (the degree to which scores reflect measurement from a ‘gold-standard’) | n/a  Sensitivity / specificity (when comparing assessment with Gold-Standard) |
| **Responsiveness** | **Responsiveness** (The ability to detect change over time in the construct to be measured) | Sensitivity/specificity (when comparing two administrations of an assessment)  Changes over time  Stability of diagnosis |
| **Interpretability** | **Interpretability** (the degree to which qualitative meaning can be assigned to quantitative scores obtained from the assessment) | n/a |

Denman, D., Speyer, R., Munro, N., Pearce, W. M., Chen, Y.-W., & Cordier, R. (2017). Psychometric properties of language assessments for children aged 4–12 years: A systematic review. *Frontiers in psychology*, *8*, 1515.

Mokkink, L. B., Prinsen, C., Patrick, D. L., Alonso, J., Bouter, L., de Vet, H. C., Terwee, C. B., & Mokkink, L. (2018). COSMIN methodology for systematic reviews of patient-reported outcome measures (PROMs). *User manual*, *78*(1).
